# Multiple mesenchymal progenitor cell subtypes with distinct functional potential are present within the intimal layer of the hip synovium

**DOI:** 10.1186/s12891-019-2495-2

**Published:** 2019-03-25

**Authors:** Asmaa Affan, Nedaa Al-Jezani, Pamela Railton, James N. Powell, Roman J. Krawetz

**Affiliations:** 1 McCaig Institute for Bone and Joint Health, Faculty of Medicine, University of Calgary, 3330 Hospital Drive NW, Calgary, Alberta T2N 4N1 Canada; 2 University of Calgary, Biomedical Engineering Graduate Program, Calgary, Canada; 3 University of Calgary, Medical Science Graduate Program, Calgary, AB Canada; 4 University of Calgary, Department of Surgery, Calgary, Alberta Canada; 5 Charles Sturt University, Orange, Australia; 6 University of Calgary, Department of Anatomy and Cell Biology, Calgary, Alberta Canada

**Keywords:** Synovial progenitor cells, Heterogeneity, Hip, Osteoarthritis, Clonal analysis

## Abstract

**Background:**

The synovial membrane adjacent to the articular cartilage is home to synovial mesenchymal progenitor cell (sMPC) populations that have the ability to undergo chondrogenesis. While it has been hypothesized that multiple subtypes of stem and progenitor cells exist in vivo, there is little evidence supporting this hypothesis in human tissues. Furthermore, in most of the published literature on this topic, the cells are cultured before derivation of clonal populations. This gap in the literature makes it difficult to determine if there are distinct MPC subtypes in human synovial tissues, and if so, if these sMPCs express any markers in vivo*/*in situ that provide information in regards to the function of specific MPC subtypes (e.g. cells with increased chondrogenic capacity)? Therefore, the current study was undertaken to determine if any of the classical MPC cell surface markers provide insight into the differentiation capacity of sMPCs.

**Methods:**

Clonal populations of sMPCs were derived from a cohort of patients with hip osteoarthritis (OA) and patients at high risk to develop OA using indexed cell sorting. Tri-differentiation potential and cell surface receptor expression of the resultant clones was determined.

**Results:**

A number of clones with distinct differentiation potential were derived from this cohort, yet the most common cell surface marker profile on MPCs (in situ) that demonstrated chondrogenic potential was determined to be CD90^+^/CD44^+^/CD73^+^. A validation cohort was employed to isolate cells with only this cell surface profile. Isolating cells directly from human synovial tissue with these three markers alone, did not enrich for cells with chondrogenic capacity.

**Conclusions:**

Therefore, additional markers are required to further discriminate the heterogeneous subtypes of MPCs and identify sMPCs with functional properties that are believed to be advantageous for clinical application.

**Electronic supplementary material:**

The online version of this article (10.1186/s12891-019-2495-2) contains supplementary material, which is available to authorized users.

## Background

Osteoarthritis (OA) is a chronic degenerative disease that is characterized by the loss of articular cartilage within the joints, resulting in inflammation and pain [1]. In regards to cell therapies in OA, and even more specifically, stem cell therapies, current studies and clinical trials have attempted to characterize mesenchymal progenitor/stem cells (MPCs/MSCs) based on their surface marker profile in order to minimalize heterogeneity of cells injected, and to provide a standardized cell based therapeutic [2–4]. However, these studies typically characterize MPC/MSC populations following in vitro culturing, and therefore it remains unknown if these cell surface markers are specific for MPCs/MSCs populations or are artifacts of cell culture [5, 6]. To our knowledge, this remains true for previous clonal studies of MPCs/MSCs, in where the cells underwent some culturing prior to clonal derivation or immuno-phenotyping. However, in the majority of these pervious clonal based studies, heterogeneity between clonal populations was still typically observed [7–11]. Therefore, we propose it is essential to examine the marker profile in situ (e.g. when isolated from tissue / before cell culture), in addition to in vitro (e.g. post cell culture); and determine if there a synovial MPC subtype exists that demonstrates an increased functional capacity (e.g. differentiation ability) that can be identified through a unique marker profile.

In patients with hip OA, resident MPC populations are found to be present in the synovial membrane as well as the synovial fluid, and these MPCs have been shown to be able to differentiate into chondrocytes [12]. Thus while Synovial MPCs have the potential to contribute to articular cartilage repair, intrinsic MPC heterogeneity has been identified as an issue in proper characterization of this cell population in other tissues [13]. Furthermore, we also need to consider heterogeneity based on joint type. Hatakeyama et al. recently demonstrated that MPCs derived from the knee and hip (of the same patient) both give rise to cells with self-renewal and differentiation potential; however, knee synovial MPCs are superior to hip derived MPCs [14]. Hence, in order to distinguish MPC subtypes present in the native synovium, it is essential to examine the cell surface marker expression since cell separation can only be undertaken on cell surface proteins in live cells. Then based on that information, isolate the cells that are best suited for a given clinical application based on functional capacity of a given subtype.

To address this knowledge gap within the field, the purpose of this study was (1) to isolate single synovial MPCs and derive clonal populations from hip synovium; (2) to determine the multipotent differentiation potential of these putative MPCs; (3) to determine which MSC/MPC cell surface markers are expressed in situ vs. in vitro, and correspond to chondrogenic differentiation capacity; (4) to validate our findings by employing the determined marker profile to identify chondrogenic enriched multipotent MPCs from a new patient cohort. We hypothesized that cell surface markers present on synovial MPCs were distinct in situ vs. in vitro and that a single and/or combination of these markers would be able to identify MPCs with increased chondrogenic potential.

## Methods

### Ethics statement

This study protocol was approved by the University of Calgary Human Research Ethics Board (REB15–0005 and REB15–0880). All participants provided written consent to participate. All testing was carried out in accordance with the declaration of Helsinki.

### Description of patients

MPCs were isolated from the synovial membrane of two groups of patients. The first were patients who had a periacetabular osteotomy (PAO) procedure done to correct acetabular dysplasia (ACD) or femoroacetabular impingement (FAI) (*n* = 12, 9 female, 3 male, average age = 25.3 years); while the second group was comprised of patients who received a total or partial hip joint replacement due to end-stage OA (*n* = 22, 9 female, 13 male, average age = 56.7 years).

### Experimental design

**Fig. 1:**
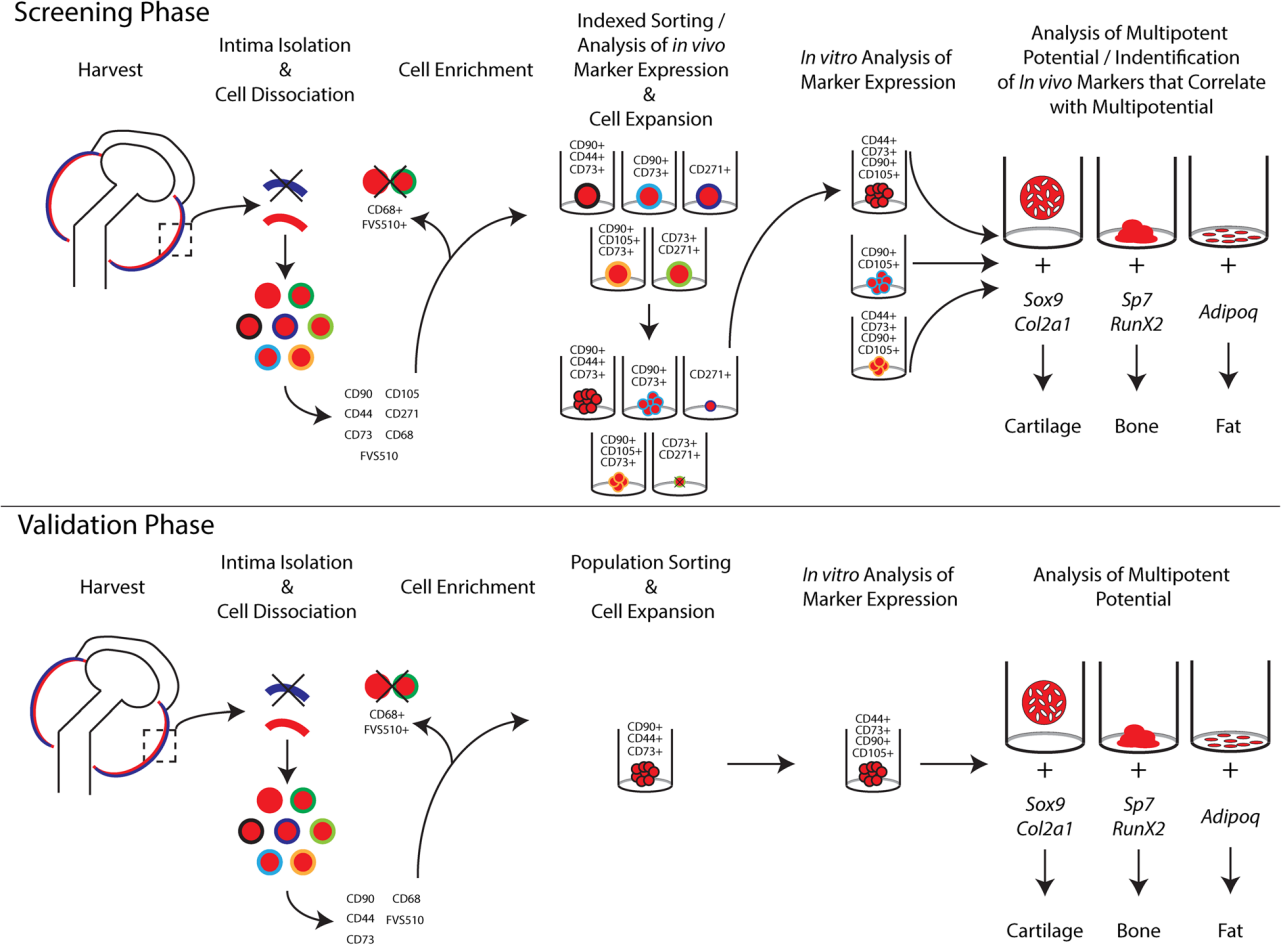
Experiment design flow chart

An overview of the experimental design of the project is presented in Fig. 1.

### Synovial membrane tissue digestion

The intimal layer was dissected from the synovial biopsies and was then cut into 5 mm^2^ pieces. It was subsequently digested in 1 mg/ml type IV collagenase (Sigma) in heat inactivated fetal bovine serum (FBS)(ThermoScientific) for 120 min at 37 °C with shaking, in order to obtain a single cell suspension. The cells were then washed with phosphate buffered saline (PBS) and immediately immunophenotyped with the International Society for Cellular Therapy (ISCT) [15] recommended MPC/MSC cell surface markers. The MSC/MPC markers included were: CD90 (Clone # 5E10, PE), CD271 (Clone # C40–1457, BV421), CD44 (Cone # G44–26, PE-Cy7), CD73 (Clone # AD2, APC), and CD105 (Clone # 266, BV650), a macrophage marker, CD68 (clone # Y1/82A, FITC), and a cell viability marker, fixable viability stain (FVS) 510 (BV510) (all BD Biosciences). UltraComp eBeads (eBioscience) individually stained with each single colour as well as unstained cells were used as compensation controls.

### Flow cytometry

The stained cells underwent fluorescent activated cell sorting (FACS) on a BD FACS Aria Fusion (BD Biosciences). Macrophages (CD68^+^) as well as the dead cells (FVS510^+^) were excluded. The remaining cells were unbiasedly (e.g. cells were not isolated based on the expression/absence of any marker / combination of markers) indexed-sorted (e.g. single cell into single well) into a 96-well plate containing 100 μL DMEM/F-12 media (Lonza- BioWhittaker) with 10% MSC stimulatory supplement (Stem Cell Technologies) with 1% antibiotic-antimycotic (ThermoFisher). The indexed-sorting recorded the presence/absence of any/all cell surface markers per cell (referred to as in situ marker data). The indexed sorting was undertaken using a “single cell” mask to reduce the chance of having multiple cells per well in addition to using a 100 μM sort nozzle and low flow rate (45% of system maximum) to reduce the pressure on the cells.

### Clonal cell expansion

The clonally derived cells within the 96-well plates were incubated at 37 °C and 5% CO_2._ Cell culture media consisted of DMEM/F-12 media (Lonza- BioWhittaker) with 10% MSC stimulatory supplement (Stem Cell Technologies) with 1% antibiotic-antimycotic (ThermoFisher). Once the cells reached ~ 70% confluency in the 96-well plate, the cells were passaged using trypsin (Corning). Cells were transferred to 12-well plates, then T25 and finally T75 flasks (all Primaria, Corning) with each successive passage.

### Differentiation

The clonal cell lines were expanded until ~ 0.75 × 10^6^ cells were obtained (~ 19 population doublings). At this point, they underwent multi-lineage differentiation analysis to determine their osteo/chondro/adipo-genic capacity.

*Osteogenesis*: For each replicate, 5 × 10^5^ cells were seeded into each well in a 24-well plate and then placed into DMEM/F-12 media that contained Dexamethasone (final concentration (FC): 100 nM) (Sigma), L-Ascorbic Acid (FC: 50 μg/mL) (Sigma), β-Glycerolphosphate (FC: 10 mM) (Sigma).

*Adipogenesis*: For each replicate, 5 × 10^5^ cells were seeded into each well in a 24-well plate and then placed into DMEM/F-12 media that contained Dexamethasone (FC: 1 μM) (Sigma), Insulin (FC: 10 μM) (Sigma), Indomethacin (FC: 200 μM) (Sigma), and Isobutylmethylxanthine (FC: 500 μM) (Sigma).

*Chondrogenesis*: For each replicate, 5 × 10^5^ cells were pelleted through centrifugation and placed into DMEM/F-12 media that contained Dexamethasone (FC: 10 nM) (Sigma), L-Ascorbic Acid (FC: 50 μg/mL) (Sigma), MEM Non-Essential Amino Acids (FC: 1%) (MEM-NEAA Gibco), Transforming growth factor (TGF)-β3 (FC: 10 ng/mL) (Peprotech), Bone morphogenetic protein (BMP)-2 (FC: 500 ng/mL) (Peprotech), insulin transferrin selenium (FC: 1%) (Lonza- BioWhittaker), and sodium pyruvate (FC: 1%) (ThermoFisher). Media was adjusted to neutral pH (7.0–7.6).

After 21 days of osteogenic, adipogenic or chondrogenic differentiation, with media changes performed twice a week, differentiation was assayed using reverse transcriptase quantitative polymerase chain reaction (RT-qPCR) and histological staining.

### RT-qPCR

mRNA was isolated using the TRIzol reagent protocol (ThermoFisher) following the manufactures instructions with the addition of glycogen solution (Amresco) to increase the yield of mRNA. Chondrogenic cultures alone went through an additional spin column step (OMEGA bio-tek E.Z.N.A. Total RNA Kit I) to remove additional ECM proteins which could potentially interfere with downstream applications. For first strand synthesis, mRNA was then added cDNA Master Mix (High Capacity cDNA kit, Applied Biosystems) following the manufactures instructions. The cDNA was stored at − 20 °C until use.

RT-qPCR analysis was employed to quantify the gene expression levels of each of the markers expressed by different lineages (osteoblasts, chondrocytes, adipocytes) as a surrogate outcome to measure the multipotent differentiation capacity of the sMPCs. For osteogenesis, gene expression of Osterix (*Sp7*) (Probe set # Mm00504574_m1) and *Runx2* (Probe set # Mm00501584_m1) were quantified. For adipogenesis, *ADIPOQ* (Probe set # Mm00456425_m1) was quantified. For chondrogenesis, *Sox9* (Probe set # Mm00448840_m1) and *Col2a* (Probe set # Mm01309565_m1) were quantified. Ribosomal 18S (Probe set # Mm03928990_g1) was employed as a housekeeping gene. All TaqMan Gene Expression Assays were obtained from Applied Biosystems. TaqMan Universal PCR Master Mix No AmpErase (Applied Biosystems) was used following the manufacturers instructions. Three replicates were run per sample and all samples were run on an ABI 7900 (Applied Biosystems) using the following program: UNG incubation - 50 °C 2 min; Enzyme activation − 95 °C 20 s; Denaturation - 95 °C 3 s; Annealing / Extending - 60 °C 30 s (40 cycles). Resulting threshold (Ct) values were analyzed using the ΔΔCt method against 18S endogenous control and undifferentiated cells as the reference sample.

### Histological staining

For further analysis of differentiation, histological staining were performed post differentiation. For osteogenic and adipogenic differentiations, the wells were fixed with 10% neutral buffered formalin (NBF) for one hour. The osteogenic wells were stained with a 0.2% Alizarin Red S (Sigma) solution in the dark for 10–15 min. The adipogenic wells were stained with a 0.5% Oil Red O solution (Sigma) for 15 min. For chondrogenic pellets, whole-mount staining was performed as follows. Pellets were fixed with 10% NBF for three hours, then washed with distilled water. The pellets were then stained with 0.1% Safranin O solution (Fisher Chemical) for 45 min in the dark. The pellets were then de-stained and transferred to PBS.

### Controls for enzymatic digestion, cell sorting, and antibody staining

To control for artefacts in the clonal MPCs induced by enzymatic digestion of the synovium, cells were plated on a 12-well plate before tissue digestion (e.g. cell outgrowth from the intact synovial tissue) to demonstrate that the tissue contained viable cells. Cells were also plated after tissue digestion in order to demonstrate that the digestion procedure did not negatively affect cell viability. And lastly, cells were plated after the immunophenotyping staining procedure (but without cell sorting) to demonstrate that the staining procedure did not reduce cell viability. The cells under all of these conditions were then allowed to proliferate under the same conditions and the same outcome procedures (e.g. differentiation analysis) were performed as the index sorted sMPCs.

### In vitro analysis of cell surface markers by flow cytometry

At the point the individual sMPC clones were ready to be placed under differentiation conditions (e.g. ~ 0.75 × 10^6^ cells) the cells were re-immunophenotyped with the same MPC markers (CD90, CD73, CD44, CD271, and CD105) and analyzed on the BD Fusion using the same settings as the indexed sorting described previously.

### Non-clonal FACS of sMPC populations

Once information regarding the cell surface markers present on clonal MPCs with chondrogenic potential was determined, this was used to isolate and expand MPCs using non-clonal FACS. Cell suspensions from 4 new patients (*n* = 2 POA, 1 female, 1 male, average age = 34.2 years) (*n* = 2 OA, 1 female, 1 male, average age = 63.1 years) were derived using methods described previously. The cell suspension was stained with CD90, CD73, CD44, CD68 and the cell viability marker FVS510. CD68^+^ and FVS510^+^ cells were excluded and then the remaining cells were sorted to obtain a purified populations of cells that were positive for all three MPC markers (CD90, CD73, and CD44). The cells were then expanded until ~ 0.75 × 10^6^ cells were obtained (~ 8 population doublings). They then underwent immuno-phenotyping by flow cytometry and differentiation analysis followed by RT-qPCR and histology as described above.

### Data analysis

For a cell surface marker to be positive for flow cytometry/FACS, the given cell (or population) had to demonstrate a fluorescence signal above the 95th percentile of the unstained/isotype control. For an mRNA lineage marker (e.g. *Sox9*, *Sp7*) to be positive in RT-qPCR analyses, there had to be a statistical increase of a significance value set at *p* < 0.05 versus the undifferentiated control (derived from the same clonal population). For a histological marker to be positive, the cells had to demonstrate a dark robust staining compared to the undifferentiated/negative control. In order for a given clonal MPC line to be considered positive for any of the three lineages tested (e.g. osteoblast, chondrocyte, adipocyte), a given MPC line had to demonstrate a positive result for both the RT-qPCR data (at least one expressed gene per lineage) in addition to the histological stain. If a cell line was only positive for RT-qPCR or histology, the cell line was considered negative for that lineage.

### Statistics

The RT-qPCR data were analyzed using GraphPad Prism 7 (GraphPad Software). The data had been reported as ± standard deviation (SD). Statistical analysis was performed with a paired t-test since the undifferentiated controls for each experiment performed are derived from the same clone as the differentiated cells. An alpha value of *p* < 0.05 was regarded as statistically significant.

## Results

### Clonal MPC derivation from patients

In this study, synovial biopsies were recovered from 34 patients undergoing orthopedic procedures of the hip (Table 1**).** From the 34 patients sampled, clonal lines were derived from 16 patients. However, only 22 MPC lines from 7 patients (4 PAO, 3 OA) demonstrated the required self-renewal potential to reach a cell population size suitable for downstream applications (e.g. differentiation and flow cytometry)(Table 1). More detailed information of the MPC lines derived from each patient is reported in Additional file 1: Table S1. To complete all the differentiation outcome measures and in vitro flow cytometry analysis (including replicates), it was determined that a clonally derived synovial MPC line must be able to go through ~ 19 population doublings. The vast majority of clonal cell lines did not display the self-renewal capacity required to achieve the level of characterization required for this study design (Table 1). In both POA and OA patients ~ 20% of the MPC clones derived demonstrated sufficient self-renewal capacity to reach at least 19 population doublings (Table 1).

**Table 1:** Description of experiments conducted on patient samples received

Description	Number of Patients (cell lines)
# patients from which biopsies were digested and sorted	34 (~ 3500 cells indexed sorted)
# of clonal cell lines that demonstrated self-renewal capacity	16 (108 cell lines)
# of cell lines that underwent at least 19 population doubling events and were included in the analysis	OA: 3 (10 cell lines)
	PAO: 4 (12 cell lines)
# of patients for deriving control populations (e.g. effect of digestions, antibody staining)	10 (10 non-clonal cell lines)

### Characterization of synovial MPC clones

**Fig. 2:**
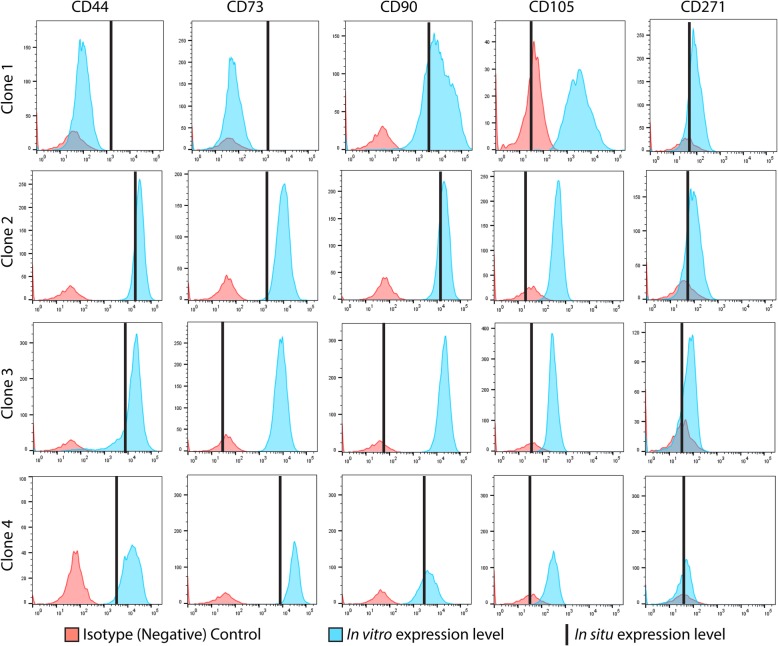
In situ and in vitro cell surface marker data from 4 clones from the same patient. The in situ expression of each CD marker is represented by the vertical bar black. The in vitro expression of each marker in the clonal derived cell population is represented by the blue histogram. The isotype/negative control for each CD marker is represented by the red histogram

Of the clonal MPC lines that demonstrated sufficient self-renewal capacity to be analyzed through differentiation and flow cytometry, the data from one patient is presented below as an example of data that was collected on all cell lines in the current study. The results for all other clonal MPC lines are summarized in Table 2. The representative data was obtained from a 47 year old female patient with hip OA. The cell surface receptor expression profile of the clonal cell lines as assayed in situ (before culture, black vertical line) and in vitro (after culture, blue histogram). Flow data are presented from four clonal cell lines (#1–4) derived from this single patient (Fig. 2). MPC clone #1 was positive for CD44, CD73 and CD90, while negative for CD105 and CD271 in situ. After expansion in culture, MPC clone # 1 retained expression of CD90, gained expression of CD105, lost expression of CD44 and remained negative for CD73 and CD271. Clone # 2 and 4 demonstrated the same profile in situ and in vitro. Specifically, in situ they demonstrated positive staining for CD44, CD73, CD90 and did not express CD105 nor CD271. In vitro they maintained expression of CD44, CD73 and CD90, remained negative for CD271 and gained CD105 expression. Clone # 3 expressed only CD44 in situ, and gained the expression of CD73, CD90 and CD105 in vitro.

**Table 2.** Summary of clonal cell lines used in the study

Patient Type	In situ Cell Surface Markers	In vitro Cell Surface Markers	Adipogenesis Histology/PCR	Chondrogenesis Histology/PCR	Osteogenesis Histology/PCR
OA	CD90,44,73	CD90,44,73,105	+/+ (positive)	+/−	−/−
OA (1)	CD90,44,73	CD90,105	+/+ (positive)	+/+ (positive)	−/−
OA (2)	CD90,44,73	CD90,44,73,105	+/+ (positive)	−/−	−/−
OA (3)	CD44	CD90,44,73,105	−/−	−/−	−/−
OA (4)	CD90,44,73	CD90,44,73,105	+/+ (positive)	−/−	−/−
OA	CD90,44,73	CD90,44,271,105	+/+ (positive)	−/+	+/+ (positive)
OA	CD90,44,73	CD90,44,73,105	+/+ (positive)	+/−	−/−
OA	CD90,44,73	CD90,44,73,105	+/+ (positive)	−/−	−/−
OA	CD90,44,73	CD90,44,73,105	+/+ (positive)	−/−	−/+
OA	CD90,44,73	CD90,44,73,105	+/+ (positive)	−/−	−/+
PAO	CD90,44,73	CD90,44,73,105	+/+ (positive)	+/+ (positive)	+/+ (positive)
PAO	CD90,44,73	CD90,44,73,105	+/+ (positive)	−/−	+/−
PAO	CD90,44,73	CD90,44,73,105	+/+ (positive)	+/+ (positive)	+/+ (positive)
PAO	CD90,44,73	CD90,44,73,105	+/+ (positive)	+/+ (positive)	−/+
PAO	CD90,44,73	CD90,44,73,105	+/−	−/−	+/+ (positive)
PAO	CD90,44,73	CD90,44,73,105	+/+ (positive)	+/−	+/+ (positive)
PAO	CD90,44,73	CD90,44,73,105	+/+ (positive)	+/+ (positive)	+/+ (positive)
PAO	CD90,44,73	CD90,44,73,105	+/+ (positive)	−/−	+/−
PAO	CD90,44,73	CD90,44,73,105	+/+ (positive)	+/−	+/+ (positive)
PAO	CD90,44,73	CD90,44,73,105	+/+ (positive)	+/+ (positive)	+/+ (positive)
PAO	CD90,44,73	CD90,44,73,105	+/+ (positive)	−/−	+/+ (positive)
PAO	CD90,44,73	CD90,44,73,105	+/+ (positive)	+/+ (positive)	+/+ (positive)

The 4 OA clones with numbers (1–4) are the clones presented throughout the results section

The + symbol represents a positive outcome of differentiation analysis. The - symbol represents a negative outcome of differentiation analysis. The word positive in brackets represents that a clonal cell line was able to differentiate into the specified lineage

**Fig. 3:**
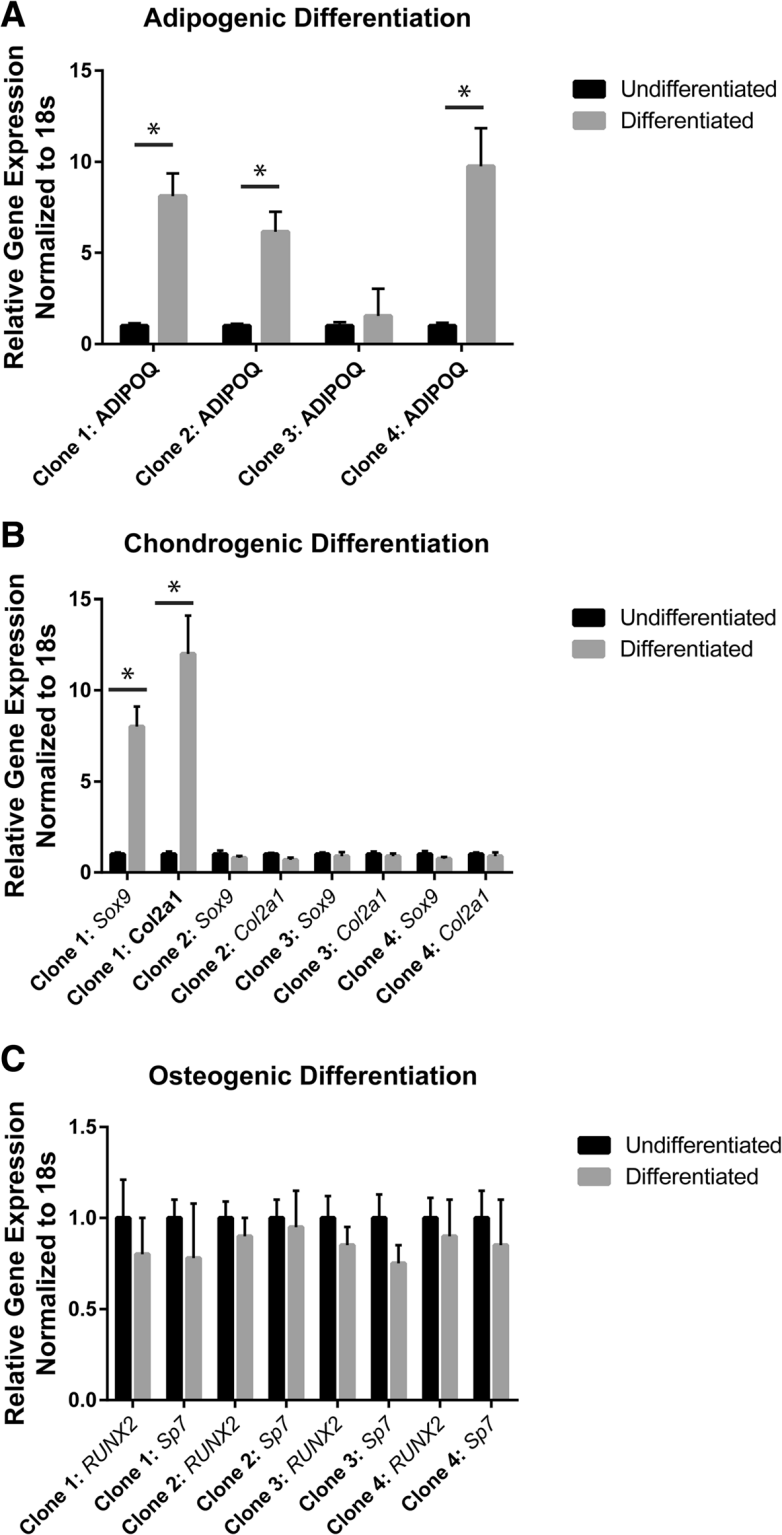
Gene expression after adipogenic (**a**), chondrogenic (**b**) and osteogenic (**c**) differentiation. Results from 4 clonal cell lines from a single OA patient. The differentiated gene expression values are normalized to undifferentiated gene expression values from the same clone. *p < 0.05

**Fig. 4:**
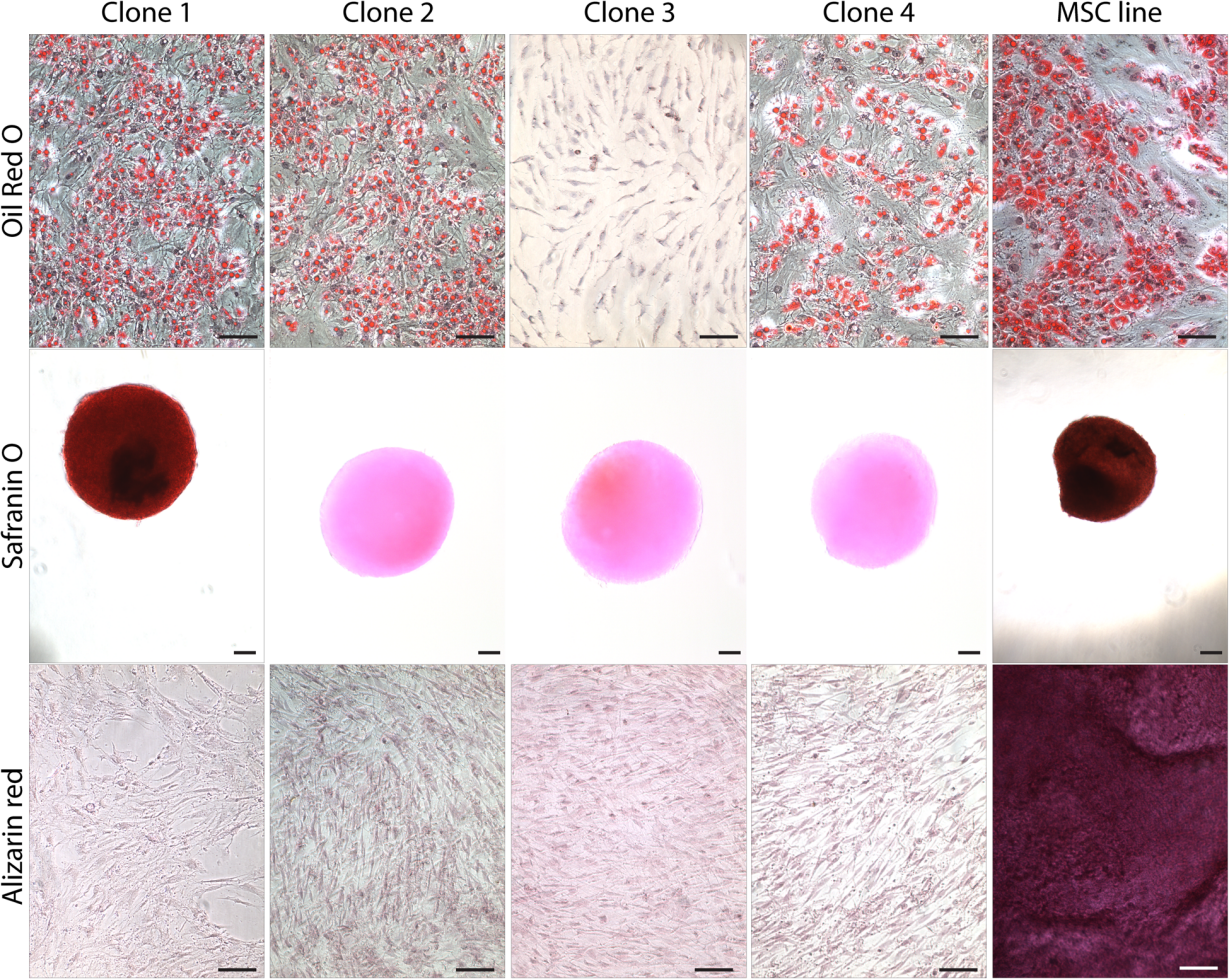
Histological analysis of differentiation. Oil Red O staining was employed to detect lipid accumulation after adipogenic differentiation (top row). All clones examined except clone # 3 demonstrated Oil Red O positive staining. Safranin O staining was employed to detect proteoglycan accumulation after chondrogenic differentiation (middle row). Only clone # 1 demonstrated positive Safranin O staining. Alizarin red staining was employed to detect calcium accumulation after osteogenic differentiation (bottom row). None of the clones examined demonstrated positive Alizarin red staining. A characterized and non-clonal MSC line was used as a positive control (right column) and demonstrated positive staining for Oil Red O, Safranin O and Alizarin Red. Scale bars equal 50 μm for Oil Red O and Alizarin Red stained images, and 200 μm for Safranin O stained images

All clonal cell lines were induced to differentiate into osteoblasts, chondrocytes and adipocytes and analyzed by RT-qPCR and histology. After the induction of adipogenesis, only clones # 1, 2 and 4 demonstrated up-regulation of *ADIPOQ* (Fig. 3a). After the induction of chondrogenesis, only clone #1 demonstrated an increase in *Sox9* and *Col2A1* expression (Fig. 3b). None of clones displayed up-regulation for the osteogenic markers *Runx2* or *Sp7* after osteogenic induction (Fig. 3c). To supplement the molecular data; histological analysis of differentiation is presented in Fig. 4. Clones # 1, 2 and 4 demonstrated positive Oil Red O staining for lipids after adipogenic differentiation. Positive staining for proteoglycans after chondrogenesis was observed only in clone #1. No Alizarin Red staining after osteogenesis in any of the 4 clones was observed (Fig. 4). Interestingly, while the molecular and histological data is in agreement for the 4 MPC clones presented from this patient; not all histological data was consistent with the molecular data in clones derived within this study (Table 2). Therefore, it was decided that a positive outcome for differentiation (into any lineage) would be based on a positive result for both the molecular and histological outcomes for differentiation (Table 2).

### Analyzing the differentiation potential of CD90^+^CD44^+^CD73^+^ MPCs

Based on the most common cell surface marker profile observed in MPCs that demonstrated chondrogenic capacity (CD90^+^CD44^+^CD73^+^); a new cohort of patients (*n* = 2 POA, *n* = 2 OA) was recruited and cell sorting (non-indexed) was performed on freshly derived synovial cell populations. CD90^+^CD44^+^CD73^+^ triple positive cells from only one OA patient demonstrated chondrogenic differentiation capacity (Table 3), and importantly, no CD90^+^CD44^+^CD73^+^ triple positive cells met the minimum criteria to be defined as MSCs. Furthermore, when the cell were re-immunophenotyped after cell culturing all 4 cell lines expressed CD44, CD73, CD90, CD105 and lacked the expression of CD271.

**Table 3.** Cell Sorting of CD90, CD44, and CD73 triple positive sMPC populations

Patients	Adipogenesis Histology/PCR	Chondrogenesis Histology/PCR	Osteogenesis Histology/PCR
POA (CD90+,44+,73+)	+/+ (positive)	−/−	+/−
POA (CD90+,44+,73+)	+/+ (positive)	−/−	−/−
OA (CD90+,44+,73+)	+/+ (positive)	+/+ (positive)	−/+
OA (CD90+,44+,73+)	−/+	−/−	−/−

Positive indicates that those cells were positive for CD90, CD44, and CD73

The + symbol represents a positive outcome of differentiation analysis. The - symbol represents a negative outcome of differentiation analysis. The word positive in brackets represents that a clonal cell line was able to differentiate into the specified lineage

## Discussion

While a number of groups worldwide are exploring the use of MSCs/MPCs for treatment of chronic disease such as OA; a recognized issue is high patient to patient variability in terms of treatment effect and cell quality/behaviour/potency [16–18]. When isolating MPCs within the same tissue from a number of patients, it is not uncommon for the resultant MPC populations to demonstrate heterogeneity in their multi-potential/differentiation abilities [19, 20]. Furthermore, between normal and diseases tissues, it has been shown that there are differences in the quantity of MPCs; their differentiation potential; and their ability for immunomodulation [20, 21]. This MPC heterogeneity may contribute to the lack of efficacy observed in many MPC clinical studies published to date. Hence, it is essential to gain a better understanding of MPC subtypes and characterize their functional capacity so that defined MPC subtypes can either be enriched for or excluded for a given therapeutic application based on their functional capacity. Therefore, defining subtypes of MPCs based on their in situ cell surface marker profile may provide a better baseline for identifying and isolating the cells best suited for chondrogenic differentiation, or at the very least a more effective way for controlling the quality/consistency of cells used for therapy.

This heterogeneity of MPC subtypes within and between patients may be responsible for the broad spectrum of results observed in this study and others. Additional file 1: Table S1 presents the population doublings of the clonal cell lines isolated and assessed. As expected, many of the clonal cell lines were found to lose self-renewal capacity at different time points throughout cell culture, and overall, very few clonal lines demonstrated sufficient self-renewal capacity to generate the number of cells required for analysis. Furthermore, a number of patient samples (from PAO and OA) did not generate any clonal populations after indexed sorting; although MPC lines (non-clonal) were able to be derived from every tissue sample as a positive control. One explanation could be that some cell subtypes may be more sensitive to the loss of cell-cell contact than others and the requirement of cell-cell contact could be different in the POA vs. OA intima. We also observed that the self-renewal capacity of MPCs both within and between patients varied greatly. One reason behind this could be attributed to cell exhaustion. It has been observed previously that stem cells may become exhausted in diseased/injured tissues and that eventually these cells may undergo replicative senescence either in vivo or in vitro [22, 23]. Since we did not have access to normal hip synovium in the current study, we were not able to test if this hypothesis had merit, but in future studies this should be examined. Furthermore, previous studies have shown that hip derived synovial MPCs are in less abundance and demonstrate inferior properties compared to knee synovial MPCs [14]. Therefore, it is possible that the reason that many MPCs underwent replicative senescence or failed to thrive in culture after isolation could be because of some inherent property specifically of hip MPCs. We are now undertaking a complementary study in knee synovial MPCs to test this hypothesis. Furthermore, without a normal synovial control population, we were not able to determine if there was any effect of the disease state and/or severity of disease on our results. As it has been shown that inflammation can affect the behaviour of MPCs/MSCs, and the level of synovial inflammation can change with disease state [24–26], it is possible that some of the heterogeneity between patients could be due to differing levels of synovial inflammation and/or other confounding variables due to disease severity.

Of the MPCs in this study that demonstrated sufficient self-renewal capacity for differentiation analysis; cells with chondrogenic potential typically expressed CD90^+^CD44^+^CD73^+^. To test if this profile discriminated for MPCs in situ with chondrogenic ability, freshly derived synovial cells were purified based on these markers and underwent differentiation analysis. However, only one out of the 4 cell lines tested demonstrated chondrogenic capacity. This suggests that CD90^+^CD44^+^CD73^+^ expression does not provide any information on the functional properties of the cells in terms of chondrogenic potential. This further indicates that these specific markers used to isolate the MPCs from synovial membranes of patients with hip dysplasia or end-stage OA of the hip joint, are insufficient to isolate the cells of interest (e.g. chondrogenic capable). What remains unknown is if the lack of functional data provided by these markers is generalizable to other MPCs from other tissues within the body (e.g. fat, bone marrow), or if this observation is only specific to cells from the hip joint.

Our current study has a number of common findings with previous clonal MSC/MPC studies performed with synovium or synovial fluid derived cells. In most of these studies, significant variability in proliferation rates between and within donors have been observed in addition to a wide range of cell potency [27–29]. Interestingly, in one study, it was observed that most clones presented with osteogenic and chondrogenic capacity, yet adipogenic potency was typically absent [30]. This is in contrast with our current study which observed the opposite trend. This could be due to methodological differences and/or tissue source (knee vs. hip synovium).

Many of the current clinical trials being undertaken use MSCs/MPCs derived from varied tissues such as bone marrow, adipose tissue, and synovial membrane. Most, if not all of these studies, isolate the cells from the tissue and either culture them and then analyze their cell surface markers after culturing, or they immediately inject the cell solution into the joint as a therapy for OA. As stated, all marker identification occurs in vitro, and they fail to characterize the cell surface markers in situ. In our study, we used known MPC markers to identify the cells in situ, and we found some interesting discrepancies. First, CD105, a known MSC/MPC marker, was only expressed on the cells once they were cultured, and were not present when the cells were analyzed in situ. This is of interest since previous groups have suggested that CD105 may in fact be an artifact of culture and is required by the cells to adhere to plastic [31, 32]. Secondly, CD271, also a known MSC/MPC marker, was not expressed on any of the MPCs isolated in situ, and was not expressed once the cells were cultured. This is of interest since CD271 expression is known to be variable in MPCs/MSCs derived from different tissues [33]. While CD271 expression has been observed in knee synovial MPCs/MSCs [26] the current study suggests that it may not be expressed on hip synovial derived cells. Overall these findings suggests that there may be issues with examining cell surface markers on MPCs only after they have been cultured; and that looking at the profiles in situ may provide a more reliable picture into marker expression on MPC subtypes within the synovial membrane.

There are some limitations in the current study. First, it is difficult to correlate the results that were observed between the different patient samples, due to the known diverse MPC phenotypic subtypes found in each patient and the overall low sample size of clones that were able to proliferate sufficiently to be examined through flow cytometry and differentiation analysis. Secondly, it was not possible to exclude synovial fibroblasts during the indexed sorting since there is no known marker that is differentially expressed between synovial fibroblasts and MPCs in situ. In vitro, both MPCs and fibroblasts have the ability to adhere to plastic and express CD90, CD44, and CD105 [34]. Therefore, some of the low clonal derivation efficiency could be due to fibroblast contamination. Hence it would be pertinent to develop additional cell surface markers as a way to isolate enriched populations of MPCs directly from tissues.

## Conclusions

In conclusion, the findings of this study indicate that there is significant heterogeneity of synovial MPC function in terms of differentiation capacity within the hip joint. Furthermore, although the cell surface marker profile in situ CD90^+^CD44^+^CD73^+^ was most commonly observed on cell with chondrogenic potential; cells expressing these 3 markers in situ do not necessarily retain chondrogenic capacity. Overall, this study aimed at paving a way for MPCs to be isolated from the hip synovial membrane based on their cell surface markers in situ as opposed to the markers that have been previously established in vitro, and we have also demonstrated that cell expansion in culture alters the surface marker profile on these MPCs. Additional studies should be undertaken to identify if these results are observed in other joints (e.g. knee) and/or other tissues of the body. If so, additional markers that are expressed on MPCs/MSCs in situ may be required so that cells can be isolated and/or enriched based on their desired functional capacity for the treatment of diseases such as OA.

## Additional file

Additional file 1: **Table S1.** Summary of all clonal lines derived from all patients included in the study. (DOCX 19 kb)

## Abbreviations

ACD: Acetabular dysplasia
FACS: Fluorescence activated cell sorting
FAI: Femoroacetabular impingement
FBS: Fetal bovine serum
ISCT: International Society for Cellular Therapy
MSCs: Mesenchymal stem cells
OA: Osteoarthritis
PAO: Periacetabular osteotomy
PBS: Phosphate buffered saline
RT-qPCR: Reverse Transcriptase quantitative polymerase chain reaction
sMPC: Synovial mesenchymal progenitor cell
