# Supplementary material for: Multiple mesenchymal progenitor cell subtypes with distinct functional potential are present within the intimal layer of the hip synovium
Source: BMC Musculoskelet Disord. 2019 Mar 25;20:125. doi: 10.1186/s12891-019-2495-2 (PMC6434889; doi:10.1186/s12891-019-2495-2)
Supplement: Supplementary file 1 — Table S1. Summary of all clonal lines derived from all patients included in the study. (DOCX 19 kb) [file 12891_2019_2495_MOESM1_ESM.docx]

**Supplementary Table 1.** Summary of all clonal lines derived from all patients included in the study.

**Grey highlight:** Clonal cell line yielded enough number of cells for full characterization.

| **Patient Type** | **Number of clones established** | **Final cell count** | **Population doublings** |
| --- | --- | --- | --- |
| PAO | 5 | 557,333 | 19.08 |
|  |  | 836,000 | 19.66 |
|  |  | <60,000 | <15.86 |
|  |  | <1000 | <9.96 |
|  |  | <1000 | <9.96 |
| PAO | 0 | - | - |
| PAO | 45 | 933,333 | 19.82 |
|  |  | 333,333 | 18.34 |
|  |  | 800,000 | 19.60 |
|  |  | 946,666 | 19.84 |
|  |  | <20,000 | <14.23 |
|  |  | <30,000 | <14.86 |
|  |  | <30,000 | <14.86 |
|  |  | <30,000 | <14.86 |
|  |  | <400,000 | <18.60 |
|  |  | <30,000 | <14.86 |
|  |  | <30,000 | <14.86 |
|  |  | <30,000 | <14.86 |
|  |  | <30,000 | <14.86 |
|  |  | <30,000 | <14.86 |
|  |  | <500,000 | <18.92 |
|  |  | <30,000 | <14.86 |
|  |  | <300,000 | <18.18 |
|  |  | <30,000 | <14.86 |
|  |  | <30,000 | <14.86 |
|  |  | <20,000 | <14.23 |
|  |  | <20,000 | <14.23 |
|  |  | <30,000 | <14.86 |
|  |  | <30,000 | <14.86 |
|  |  | <30,000 | <14.86 |
|  |  | <30,000 | <14.86 |
|  |  | <20,000 | <14.23 |
|  |  | <30,000 | <14.86 |
|  |  | <30,000 | <14.86 |
|  |  | <20,000 | <14.23 |
|  |  | <30,000 | <14.86 |
|  |  | <30,000 | <14.86 |
|  |  | <30,000 | <14.86 |
|  |  | <30,000 | <14.86 |
|  |  | <20,000 | <14.23 |
|  |  | <20,000 | <14.23 |
|  |  | <30,000 | <14.86 |
|  |  | <20,000 | <14.23 |
|  |  | <20,000 | <14.23 |
|  |  | <30,000 | <14.86 |
|  |  | <30,000 | <14.86 |
|  |  | <20,000 | <14.23 |
|  |  | <100,000 | <16.60 |
|  |  | <60,000 | <15.86 |
|  |  | <60,000 | <15.86 |
|  |  | <20,000 | <14.23 |
| PAO | 1 | 720,000 | 19.45 |
| PAO | 0 | - | - |
| PAO | 0 | - | - |
| PAO | 0 | - | - |
| PAO | 0 | - | - |
| PAO | 0 | - | - |
| PAO | 0 | - | - |
| PAO | 0 | - | - |
| PAO | 8 | <30,000 | <14.86 |
|  |  | 1,533,333 | 20.54 |
|  |  | 2,056,667 | 20.96 |
|  |  | 3,253,333 | 21.62 |
|  |  | 630,000 | 19.25 |
|  |  | 2,803,333 | 21.41 |
|  |  | <300,000 | <18.18 |
|  |  | 2,453,333 | 21.21 |
| OA | 9 | 1,200,000 | 20.18 |
|  |  | 2,933,333 | 21.47 |
|  |  | 2,933,333 | 21.47 |
|  |  | 2,066,666 | 20.97 |
|  |  | <30,000 | <14.86 |
|  |  | <30,000 | <14.86 |
|  |  | <30,000 | <14.86 |
|  |  | <30,000 | <14.86 |
|  |  | <30,000 | <14.86 |
| OA | 1 | <300,000 | <18.18 |
| OA | 14 | <60,000 | <15.86 |
|  |  | <60,000 | <15.86 |
|  |  | <60,000 | <15.86 |
|  |  | <60,000 | <15.86 |
|  |  | <60,000 | <15.86 |
|  |  | <60,000 | <15.86 |
|  |  | <60,000 | <15.86 |
|  |  | <60,000 | <15.86 |
|  |  | <60,000 | <15.86 |
|  |  | <30,000 | <14.86 |
|  |  | <60,000 | <15.86 |
|  |  | <60,000 | <15.86 |
|  |  | <60,000 | <15.86 |
|  |  | <30,000 | <14.86 |
| OA | 2 | <30,000 | <14.86 |
|  |  | 13,100 | 13.67 |
| OA | 0 | - | - |
| OA | 0 | - | - |
| OA | 6 | <30,000 | <14.86 |
|  |  | 400 | 8.64 |
|  |  | 29,100 | 14.82 |
|  |  | 764,000 | 19.53 |
|  |  | 871,000 | 19.72 |
|  |  | 588,000 | 19.15 |
| OA | 1 | 575 | 9.16 |
| OA | 3 | <1000 | <9.96 |
|  |  | <60,000 | <15.86 |
|  |  | <60,000 | <15.86 |
| OA | 0 | - | - |
| OA | 0 | - | - |
| OA | 0 | - | - |
| OA | 1 | 85,000 | 16.37 |
| OA | 0 | - | - |
| OA | 0 | - | - |
| OA | 0 | - | - |
| OA | 1 | <300,000 | <18.18 |
| OA | 8 | <30,000 | <14.86 |
|  |  | 449,333 | 18.77 |
|  |  | <60,000 | <15.86 |
|  |  | <60,000 | <15.86 |
|  |  | <60,000 | <15.86 |
|  |  | 686,667 | 19.38 |
|  |  | 990,000 | 19.91 |
|  |  | 1,336,667 | 20.34 |
| OA | 2 | <300,000 | <18.18 |
|  |  | <60,000 | <15.86 |
| OA | 0 | - | - |
| OA | 1 | 657,334 | 19.32 |
| OA | 0 | - | - |
